# Belief in a Just World and Mental Toughness in Adolescent Athletes: The Mediating Mechanism of Meaning in Life

**DOI:** 10.3389/fpsyg.2022.901497

**Published:** 2022-06-10

**Authors:** Siyu Tian, Si Chen, Yuming Cui

**Affiliations:** ^1^School of Physical Education, Shandong University, Jinan, China; ^2^School of Nursing and Rehabilitation, Cheeloo College of Medicine, Shandong University, Jinan, China; ^3^Shandong Sports Rehabilitation Research Center, Jinan, China

**Keywords:** just world theory, life meaning theory, mental toughness, athletes, positive psychology

## Abstract

Mental toughness is an essential component of adolescent athletes' athletic careers and lives. Evidence supports the positive effect of belief in a just world on individual psychological development, but the relationship between belief in a just world and mental toughness of adolescents has not been tested. In order to determine the influencing factors of mental toughness and explore effective strategies for improving adolescent athletes' mental toughness, this study introduced just world and life meaning theories to explore the relationship between belief in a just world, meaning in life (search for meaning/presence of meaning), and mental toughness. Based on the data of 1,544 adolescent athletes from Yantai and Qingdao in Shandong Province, China, we tested a parallel mediation model that considered the search for meaning and presence of meaning as mediators. The results were predicted as follows: there is a significant positive correlation between belief in a just world and mental toughness, while the relationship between belief in a just world and mental toughness was partially mediated by the search for meaning and the presence of meaning in life. Furthermore, it is worth noting that the presence of meaning played a more influential role than the search for meaning. The results suggest that belief in a just world is connected to the mental toughness of adolescent athletes *via* the meaning in life. Therefore, maintaining and promoting the level of belief in a just world and enhancing the sense of meaning in life may be an effective strategy to develop the mental toughness of adolescent athletes. The findings of this study can help develop the mental toughness of adolescent athletes and help them maintain a high level of subjective and objective performance under the pressure of training and competition, providing practical guidance for coaches and administrators in the training of adolescent athletes.

## Introduction

With rapid development in social media and communication technology, the impact of competitive sports is also expanding (Yan, 2020). The mission of athletes is not only limited to the pursuit of excellent sports performance in order to obtain honors in athletic competitions but also to shoulder responsibility for enhancing the appeal of sports culture and demonstrating to the public the spiritual qualities of competitive sports such as perseverance, optimism, indomitable struggle, and never giving up. Thus, in order to enable athletes to better express multiple values brought by sports and spread the charm of competitive sports, it is of great practical significance to discuss how to improve the positive psychological characteristics of athletes.

### Mental Toughness of Adolescent Athletes

A high level of competitive performance and cultural symbols in line with social expectations such as the spirit of perseverance and self-challenge are the basis for athletes to prove their competitive, hard power and give full play to their soft power of sports. Mental toughness is a unique concept developed in sports psychology (Guillén and Laborde, 2014). It refers to a psychological advantage whereby athletes can maintain a high level of concentration and confidence, firmly pursue personal goals when facing stressful situations, overcome adversity, and even demonstrate better sports performance under challenging circumstances (Gucciardi et al., 2015). Previous research studies have illustrated that mental toughness not only is a positive factor in promoting athletes' competitive performance but also exerts a positive influence on the psychological states of athletes, which can stimulate their enthusiasm and investment in training and enable them to avoid burnout (Lou et al., 2014; Cowden, 2016; Ye et al., 2016; Madigan and Nicholls, 2017). Adolescence is a critical stage of life in terms of individuals' overall development (Arnett, 1999). Psychological resources cultivated by athletes during their youth can help them better face pressures and dilemmas in their future sports careers, laying a solid foundation for their own sports career development (Chen et al., 2015).

Because the connotation of mental toughness is consistent with the public's impression of the cultural symbols of athletes, mental toughness is not only a critical psychological quality for athletes but also an authoritative term used to express the psychological quality of athletes (Gucciardi et al., 2015). Adolescent athletes with a high level of mental toughness can show qualities integral to the spirit of sports such as “integrity” and “fairness” and demonstrate the sports culture to society. Therefore, either from the perspective of maintaining and supporting the sports career and mental health of adolescent athletes or from the perspective of carrying forward the spirit of the sports culture, it is essential to explore the factors and mechanisms that affect the development of adolescent athletes' mental toughness and provide a theoretical basis for improving adolescent athletes' mental toughness.

Due to the significance of mental toughness to athletes, numerous studies have explored its influencing factors. Nicholls et al. (2009) found that athletes' mental toughness differs significantly according to gender, age, and sports experience. Lou et al. (2014) pointed out that team culture, social support, motor ability, coping strategy, and stress-hardy personality are important factors affecting Chinese athletes' mental toughness. Based on the basic psychological needs theory, Mahoney et al. (2014) found that the satisfaction of basic psychological needs was significantly related to the level of mental toughness of young athletes. However, although these studies partially revealed the influencing factors of athletes' mental toughness, no research has been conducted on athletes' mental toughness from the perspective of a personal belief system. As a basic component of individuals' values, the belief system has an important impact on their cognitive process and behavioral response (Yang, 2006), and mental toughness is a positive embodiment of these two aspects. Therefore, research exploring the relationship between personal belief and athletes' mental toughness is necessary, which is still lacking within the mental toughness scholarship.

### Belief in a Just World and Mental Toughness

Belief in a just world refers to people believing that the world they live in is a just world where everyone gets what they deserve (Lerner and Miller, 1978). The personal resource hypothesis of just-world belief holds that just-world belief is a positive psychological resource, which plays a role in individual cognitive bias (Wu and Li, 2014). Many studies have linked belief in a just world to positive mental health, indicating that belief in a just world is a positive and robust coping mechanism (Furnham, 2003). People with a firm belief in a just world are prone to rationalize their experiences and, as a result, respond to life's problems in a more positive way and are more likely to make sustained efforts toward achieving their goals (Kong et al., 2021). Otto and Schmidt (2007) pointed out that the adaptive psychological function of belief in a just world can provide individuals with trust in their efficacy and social environment, thus compensating for stress in the workplace and enabling them to perform better at work and exhibit a low state of exhaustion. It is noteworthy that this positive effect is very similar to the effect of individuals' increased mental toughness. However, the relationship between belief in a just world and mental toughness has not been directly examined. Moreover, adolescent athletes belong to a particular population (Chen et al., 2015); they differ from ordinary people in terms of lifestyle, living environment, and the events and pressure they need to cope with. It is necessary to test whether belief in a just world is associated with adolescent athletes' mental toughness.

### Mediation Effects of Meaning in Life

The concept of meaning in life is the individuals' perception of their self-existence and importance, as well as their understanding, the pursuit of their purpose, and the value of life (Heintzelman and King, 2016). Frankl (1963) believed that the pursuit of the sense of meaning of life is a natural psychological process found in everyone and is the most basic primitive motivation of humankind. According to the meaning-making model proposed by Park and George (2013), beliefs constitute the core schema of individuals' interpretation of life experience, which form an essential basis for individuals to develop their own unique and relatively stable experience of the meaning of life. Belief in a just world can lead people to have a more positive self-evaluation and can provide internal motivation for individuals to pursue their long-term goals, which is helpful for experiencing the meaning of life (Dalbert, 1999; Hafer, 2000). Meaning in life can help people realize the value of their existence, have a higher sense of self-efficacy and correct attribution, face various challenges with a positive attitude, and continue to strive toward achieving their goals in times of difficulty (Heintzelman and King, 2014). Therefore, it can be inferred that belief in a just world and a sense of meaning in life can serve as unique internal resources to meet the needs of athletes in training, competition, and life, thus giving them a higher level of mental toughness.

Steger et al. (2006) pointed out that the sense of meaning in life includes two independent dimensions: the search for meaning and the presence of meaning. The search for meaning reflects an individual's motivation and direction to search for meaning in life, while the presence of meaning represents a person's subjective feeling of a meaningful life (Steger et al., 2006). The search for meaning focuses on motivation and process, concerning the active degree of drive to find meaning in one's life; the presence of meaning emphasizes experiences and results, focusing on individuals' understanding and perception of their life goals and mission (Steger et al., 2006). Because the two dimensions have different emphases, the relationship between them remains uncertain (Steger et al., 2008). Previous studies have also shown that the search for meaning and the presence of meaning have different effects on the psychological characteristics of individuals (Yek et al., 2017). Therefore, the current study investigated the mediating effects of the two dimensions of meaning in life on the relationship between belief in a just world and adolescent athletes' mental toughness, respectively.

### Present Study

In sum, based on the aforementioned aspects, it is evident that the mental toughness of athletes has received an increasing level of attention, and the adolescent period is an important stage of individual psychological development. Although many studies have tested the hypothesis that belief in a just world and meaning in life are important psychological resources for individuals, the relationship between belief in a just world, meaning in life, and the mental toughness of adolescent athletes has still not been examined. Therefore, the present study proposed the following three hypothesis to examine the relationship between belief in a just world and adolescent athletes' mental toughness, and the mediating role of meaning in life. This study aimed to provide a theoretical basis for further explaining the mechanism of belief in a just world on adolescent athletes' mental toughness, and provide ideas for the development and intervention of adolescent athletes' mental toughness.

Hypothesis I: Belief in a just world has a positive relation with adolescent athletes' mental toughness.

Hypothesis II: The search for meaning in life has a mediating effect between belief in a just world and adolescent athletes' mental toughness.

Hypothesis III: The presence of meaning in life has a mediating effect between belief in a just world and adolescent athletes' mental toughness.

## Materials and Methods

### Participants and Data Collection Procedures

A total of 1,578 adolescent athletes were recruited to participate in this study to complete paper questionnaires. They were recruited by contacting the principals and managers of five sports schools and sports project management centers in Yantai city and Qingdao city of Shandong Province following cluster sampling. Sports schools and sports project management centers are the official institutions for the management of adolescent athletes. We took the full completion of the questionnaire as the standard and excluded 34 responses that did not meet the requirements. The final sample size used for analysis was 1,544, with an effective rate of 97.85%. The final sample consisted of 852 male adolescent athletes and 692 female adolescent athletes, with a mean age of 14.43 (*SD* = 1.92) and an age range of 10–19 years. The study enrolled athletes of 22 sports, including basketball, volleyball, football, athletics, swimming, rugby, weightlifting, judo, shooting, and rock climbing. Among them, 1,130 athletes participated in individual sports, and 414 athletes participated in team sports. Based on the criteria of the Chinese State General Administration of Sports, there were 12 master sportsman athletes, 138 first-level athletes, 366 second-level athletes, and 1,028 non-level athletes. Among them, 640 young athletes had changed their sports project.

With the approval of the university research ethics committee, all participants were fully informed about the purpose and methodology of the study and were given the right to agree or refuse to participate before filling in the questionnaires.

### Instruments

#### Just World Belief Scale

Personal belief in a just world was measured by the Chinese version of the Just World Belief Scale compiled by Dalbert (1999) and revised by Su et al. (2012), which consists of 13 items. A six-point Likert scale was used to measure the responses (ranging from 1 “strongly disagree” to 6 “strongly agree”). The responses to the 13 questions were summed to calculate the Just World Belief Scale score. The higher the scale score, the stronger the belief in a just world. In the present study, Cronbach's α coefficient value for the Just World Belief Scale was 0.888.

#### Meaning in Life Questionnaire

The sense of meaning in life was measured by the Chinese version of the Meaning in Life Questionnaire compiled by Steger et al. (2006) and revised locally by Liu and Gan (2010). This scale comprises two sub-scales: search for meaning and presence of meaning. Each of the sub-scales is measured by five items. The items are rated using a seven-point Likert scale, ranging from 1 “absolutely untrue” to 7 “absolutely true.” One of the 10 items is a reverse scoring question, in which the item is reversed before summing the scores. The higher the sub-scale score, the higher the degree of meaning in life. Cronbach's α coefficient was 0.775 for the total scale, 0.772 for the search for meaning sub-scale, and 0.809 for the presence of meaning sub-scale.

#### Mental Toughness Inventory

Mental toughness was measured using the Chinese version of the Mental Toughness Inventory compiled by Gucciardi et al. (2016). The scale also showed good reliability and validity in the study of Chinese athletes (Fan and Wang, 2020). In the scale, mental toughness is conceptualized as a unidimensional construct, and eight items are used to obtain the Mental Toughness Index. Participants responded to each item on a seven-point Likert scale (1 = “false 100% of the time” to 7 = “true 100% of the time”). The eight items were summed to obtain the scale's total score, and higher scores denoted a more robust mental toughness. Cronbach's α coefficient value for the Mental Toughness Inventory was 0.907.

### Statistical Analysis

The original data were inputted and checked using Excel, while SPSS 24.0 (IBM, Armonk, NY, USA) was used for statistical analysis. Descriptive statistical tests include calculating the mean and standard deviation to describe the distribution of variables. Pearson's correlation was used to calculate the correlation between each pair of variables, while independent *t*-tests and chi-square tests were used to test the group differences among different demographic categories. Hayes (2018) PROCESS macro (version 3.0), based on regression analysis, was used to conduct mediation analysis with demographic variables as covariates, belief in a just world as an independent variable, mental toughness as the dependent variable, and search for meaning and presence of meaning as mediating variables. Based on 5000 bootstrapping samples, direct, indirect, and total effects and the difference between the two mediation effects were calculated using PROCESS, and 95% bias-corrected confidence intervals (CIs) were estimated. The CIs did not contain zero when direct or indirect effects were considered significant. The significance level of all variables was set to α = 0.05. Before entering the mediation model, all variables were standardized.

## Results

### Common Method Bias Testing

Harman's single-factor test was employed to estimate the common method bias. By taking the three questionnaires into an exploratory factor analysis and examining the unrotated factor solution, we found that there were five factors with eigenvalues >1, and the first factor could account for 28.726% covariance among the measures, less than the critical value of 40% (Tang and Wen, 2020). The results demonstrated that there was no significant issue with common method bias in the current study.

### Correlation Analysis of Adolescent Athletes' Belief in a Just World, Meaning in Life, and Mental Toughness

Table 1 presents the means, standard deviations, and a correlation matrix for belief in a just world, meaning in life, and mental toughness. Belief in a just world was positively correlated with the mental toughness of adolescent athletes. The total score of the Meaning in Life Questionnaire and its dimensions was positively correlated with the belief in a just world and mental toughness of adolescent athletes. A significant correlation between variables (*P* < 0.01) provided a better foundation for the subsequent research hypothesis and mediation testing.

**Table 1:** Descriptive statistics and correlation matrix for belief in a just world, meaning in life, and mental toughness in adolescent athletes.

	***M***	***SD***	**1**	**2**	**3**	**4**	**5**
1. Belief in a just world	56.400	9.978	1				
2. Meaning in life	52.422	9.030	0.386^**^	1			
3. Search for meaning	26.137	5.678	0.210^**^	0.768^**^	1		
4. Presence of meaning	26.286	5.918	0.387^**^	0.789^**^	0.212^**^	1	
5. Mental toughness	43.186	7.885	0.406^**^	0.521^**^	0.242^**^	0.563^**^	1

*M, mean; SD, standard deviation;*

** *P < 0.01*.

### The Association of Demographic Variables With Belief in a Just World, Meaning in Life, and Mental Toughness in Adolescent Athletes

Table 2 shows the association of demographic variables with adolescent athletes' belief in a just world, meaning in life, and mental toughness. The demographic information collected included age, gender (male/female), athletic level (master sportsman/first-level/second-level/non-level), training experience (years), sports project (individual event/team event), and whether the sports project was changed or not. The results indicated that age was negatively correlated with adolescent athletes' belief in a just world, presence of meaning, and mental toughness. Female adolescent athletes demonstrated significantly lower values than male adolescent athletes for belief in a just world, meaning in life, and mental toughness. Compared to the non-level athletes, the master sportsman and first-level athletes showed a greater search for meaning. However, there was no significant difference between the master sportsman and first-level athletes (*P* > 0.05). Adolescent athletes with more training experience had a stronger perceived meaning in life, mainly in the presence of meaning. Adolescent athletes who changed their sports projects showed lower levels of belief in a just world.

**Table 2:** Associations of demographic variables with belief in a just world, meaning in life, and mental toughness in adolescent athletes.

**Variables**	**Age**	**Gender**		**Athletic level**				**Training experience (years)**	**Sports project**		**Sports project changed**	
	***r***	**Male**	**Female**	**Master sportsman**	**First-level**	**Second-level**	**Non-level**	***r***	**Individual event**	**Team event**	**Yes**	**No**
		**Mean (*SD*)**	**Mean (*SD*)**	**Mean (*SD*)**	**Mean (*SD*)**	**Mean (*SD*)**	**Mean (*SD*)**		**Mean (*SD*)**	**Mean (*SD*)**	**Mean (*SD*)**	**Mean (*SD*)**
Belief in a just world	−0.158^**^	57.180^***^	55.441^***^	55.500	55.116	56.500	56.548	−0.043	56.302	56.669	55.414^***^	57.098^***^
		(9.715)	(10.217)	(14.835)	(9.998)	(10.517)	(9.711)		(56.669)	(10.389)	(9.927)	(9.960)
Meaning in life	−0.048	53.302^***^	51.340^***^	57.083	52.471	52.527	52.324	0.066^**^	52.265	52.850	52.466	52.394
		(8.757)	(9.247)	−11.966	(9.852)	(8.783)	(8.963)		(8.942)	(9.263)	(8.986)	(9.065)
Search for meaning	−0.004	26.523^**^	25.660^**^	29.500^*^	26.587^*^	25.948	26.104	0.046	26.096	26.249	26.419	25.937
		(5.751)	(5.554)	(5.179)	(5.279)	(6.118)	(5.564)		(5.618)	(5.844)	(5.680)	(5.672)
Presence of meaning	−0.069^**^	26.778^***^	25.679^***^	27.583	25.884	26.579	26.220	0.056^*^	26.170	26.601	26.047	26.455
		(5.773)	(6.040)	(7.609)	(6.071)	(6.127)	(5.801)		(5.980)	(5.738)	(6.132)	(5.758)
Mental toughness	−0.078^**^	43.884^***^	42.327^***^	41.833	42.942	43.464	43.135	0.015	43.049	43.560	43.163	43.202
		(7.896)	(7.791)	(10.624)	(7.350)	(7.786)	(7.961)		(7.926)	(7.769)	(8.073)	(7.753)

*The Pearson correlation coefficient evaluated the correlation of age and training experience with the variables. The independent t-test was used to compare the variable differences between gender, sports project (individual event, team event), and whether the sports project was changed. The chi-square test was used to compare the variable differences between athletic levels. Multiple comparisons were made to test for differences in athletic levels with non-level athletes as the standard*.

* *P < 0.05;*

** *P < 0.01;*

*** *P < 0.001*.

### The Regression Analysis of Belief in a Just World, Meaning in Life, and Mental Toughness in Adolescent Athletes

**Figure 1:**
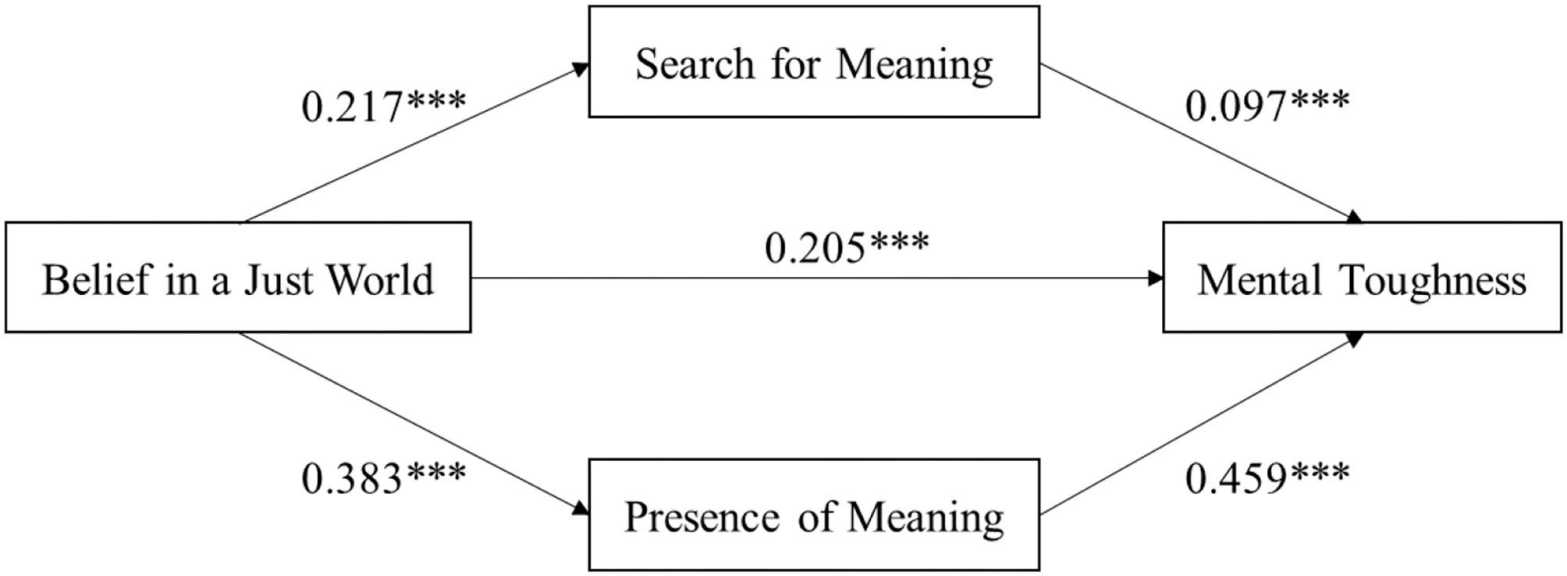
Mediation effect analysis of meaning in life between belief in a just world and mental toughness in adolescent athletes. ***P < 0.001. Demographic variables as covariances.

Table 3 shows the regression coefficients of the model established with belief in a just world as the independent variable, mental toughness as the dependent variable, search for meaning and presence of meaning as two kinds of meaning in life as the mediating variables, and demographic variables (age, gender, athletic level, training experience, sports project, and sports project changed) as control variables. The findings demonstrate that the regression coefficient was statistically significant (β = 0.402, *P* < 0.001) in the relationship between belief in a just world and mental toughness. In addition, with belief in a just world as the independent variable, and search for meaning (β = 0.217, *P* < 0.001) and presence of meaning (β = 0.383, *P* < 0.001) as dependent variables, the regression coefficients were statistically significant. Moreover, taking belief in a just world, search for meaning, and presence of meaning as independent variables and mental toughness as the dependent variable, the regression coefficients of belief in a just world (β = 0.205, *P* < 0.001), search for meaning (β = 0.097, *P* < 0.001), and presence of meaning (β = 0.459, *P* < 0.001) were also statistically significant. These results indicate that belief in a just world could positively connect to mental toughness, search for meaning, and presence of meaning in adolescent athletes. The search for meaning and the presence of meaning could also positively connect to the mental toughness of adolescent athletes. The regression weights of the path analysis are shown in Figure 1.

**Table 3:** Regression analysis of belief in a just world, meaning in life, and mental toughness in adolescent athletes.

**Outcome variables**	**Predictive variables**	**Goodness-of-fit indices**			**Regression coefficient and significance**	
		***R***	***R** ^**2**^*	***F***	**β**	***t***
Mental toughness		0.418	0.175	46.408^***^		
	Belief in a just world				0.402	16.903^***^
Search for meaning		0.2411	0.0581	13.537^***^		
	Belief in a just world				0.217	8.533^***^
Presence of meaning		0.406	0.1649	43.316^***^		
	Belief in a just world				0.383	16.008^***^
Mental toughness		0.609	0.3704	100.292^***^		
	Belief in a just world				0.205	9.029^***^
	Search for meaning				0.097	4.597^***^
	Presence of meaning				0.459	20.539^***^

*Demographic variables as covariances*.

*** *P < 0.001*.

### The Mediation Analysis of Meaning in Life Between Belief in a Just World and Mental Toughness in Adolescent Athletes

As shown in Table 4, a bias-corrected bootstrap method with 5,000 samples was conducted to estimate the mediating effects of meaning in life (search for meaning and presence of meaning) on adolescent athletes' mental toughness. The results show that the effect of belief in a just world on mental toughness was significant (direct effect = 0.205; 95% CI: 0.161, 0.250), while that on meaning in life was a significant mediator between belief in a just world and mental toughness (total indirect effect = 0.197; 95% CI: 0.166, 0.230). To be specific, the search for meaning played a mediating role between belief in a just world and mental toughness (indirect effect = 0.021; 95% CI: 0.011, 0.033); the presence of meaning played a mediating role between belief in a just world and mental toughness, and the mediating effect was 0.176 (95% CI: 0.147, 0.207). The bootstrapped 95% CI in both did not include zero, confirming the mediating effects for the association between belief in a just world and mental toughness through the search for meaning and the presence of meaning. Furthermore, in the relationship between belief in a just world and mental toughness, the mediating effect of the presence of meaning was significantly greater than that of the search for meaning (the mediating effect of the search for meaning minus the mediating effect of the presence of meaning = −0.155; 95% CI: −0.188, −0.123). These results illustrate that meaning in life played a partial mediating role between the belief in a just world and mental toughness of adolescent athletes. Moreover, compared to the search for meaning, the presence of meaning had a more substantial relation on the belief in a just world to the mental toughness of adolescent athletes.

**Table 4:** Mediation analysis of meaning in life between belief in a just world and mental toughness in adolescent athletes.

**Effect**	**Standardized coefficient**	**Bootstrap *SE***	**Bootstrap 95% CI lower limit**	**Bootstrap 95% CI upper limit**	**RME (%)**
Total effect	0.402	0.024	0.355	0.449	
Direct effect	0.205	0.023	0.161	0.250	51.00%
Total indirect effect	0.197	0.016	0.166	0.230	49.00%
BJW → SM → MT	0.021	0.006	0.011	0.033	5.22%
BJW → PM → MT	0.176	0.015	0.147	0.207	43.78%
C1	−0.155	0.016	−0.188	−0.123	

*BJW, belief in a just world; SM, search for meaning; PM, presence of meaning; MT, mental toughness; C1, the mediating effect of the search for meaning minus the mediating effect of the presence of meaning; RME, relative mediation effect*.

## Discussion

### Group Differences of Belief in a Just World, Meaning in Life, and Mental Toughness in Adolescent Athletes

Age was negatively associated with belief in a just world, presence of meaning, and mental toughness of adolescent athletes. Perhaps as athletes grow older and gain more life experience, they begin to reflect on the question of a just world. The cruelty of competitive sports and the emphasis on “geniuses” have intensified the doubts of adolescent athletes about whether the world really is just (Lacerda and Mumford, 2010). Moreover, with increasing physiological age, athletes' performance will reach a “plateau,” which makes it difficult to achieve significant progress (Corso, 2018). At this time, they may feel that they have invested too much in sports and do not have much opportunity to develop other life skills (Sun et al., 2020). This situation makes adolescent athletes worry that they are not equipped to face life away from competitive sports. Therefore, this reduces the presence of meaning for adolescent athletes and makes it difficult for them to maintain a higher level of mental toughness. The finding that male adolescents are superior to female adolescents in their belief in a just world, meaning in life, and mental toughness may be attributed to the fact that female adolescents are more modest when evaluating themselves (Rudman, 1998). In addition, in current society, the competitive sports context is often associated with masculinity (Clément-Guillotin and Fontayne, 2011). This stereotype results in male adolescent athletes receiving more social support. Thus, they will demonstrate a higher level of positive psychological resources.

The results show that compared with the non-level athletes, the master sportsman and first-level athletes have a higher level of search for meaning, which indicates that the search for meaning may be an essential psychological driving force underlying the difference between elite and ordinary athletes. Moreover, athletes with long training experience invest more time and energy in competitive sports and encounter more difficulties. In order to avoid cognitive dissonance caused by a conflict between pain and persistence of training in competitive sports (Festinger and Carlsmith, 1959), they are more inclined to think that sports give meaning to their lives. Furthermore, adolescent athletes who have changed sports projects may feel abandoned by the team and think that the world is unjust.

### Impact of Belief in a Just World on Mental Toughness

The results also show that belief in a just world was positively associated with mental toughness. Adolescent athletes with a stronger belief in a just world have higher levels of mental toughness, which is consistent with findings of previous studies on just-world beliefs and individuals' positive psychological qualities (Xiao et al., 2015; Hafer et al., 2020). Belief in a just world can promote individuals to rationalize the adverse events they encounter and reduce their sense of injustice. At the same time, people with a strong belief in a just world are less inclined to ask why they have to suffer injustice to reduce the negative impact of rumination on negative thoughts on their psychological state (Dalbert, 2002). In addition, the belief in living in a just world helps people build a sense of control, enables them to face difficulties in stressful situations, and motivates them to pursue their long-term goals (Tian, 2019; Igou et al., 2021). Therefore, adolescent athletes who firmly believe that the world is just often adopt more positive behavior and mentality to deal with difficulties in training and competition and will not be easily discouraged in the face of setbacks, enabling them to establish stronger mental toughness.

### Mediating Role of Meaning in Life Between Belief in a Just World and Mental Toughness

The study has also shown that the search for meaning and the presence of meaning played parallel mediating roles between belief in a just world and mental toughness. The results suggest that belief in a just world was positively associated with meaning in life for adolescent athletes. This is consistent with the viewpoint of Igou et al. (2021), which suggested that people who believe in a just world have a greater sense of certainty and belonging, and the need for these two senses is closely linked to the basic need for meaning in life. Thus, when belief in a just world increases, people's sense of meaning in life is enhanced. Meanwhile, another result from the current study is that meaning in life was positively associated with adolescent athletes' mental toughness. This result supports the interpretation of a previous study, where the experience of meaning in life was an important factor in the optimization of individual psychological function in emerging adults (Soucase et al., 2021). Based on the aforementioned aspects, belief in a just world appears to be related to the meaning in life (search for meaning and presence of meaning), which contributes to mental toughness.

The critical finding of this study was that there were significant differences between the search for meaning and the presence of meaning in the mediating effect between belief in a just world and mental toughness. Specifically, the presence of meaning had a significantly more positive association with mental toughness than the search for meaning. This confirmed the finding of Steger et al. (2006), who viewed the search for meaning and the presence of meaning as two independent dimensions of meaning in life, with different emphases. The result is also similar to that of a previous study, which reported that the presence of meaning was related to higher levels of positive effects, whereas the search for meaning was not (Barnett et al., 2018). Therefore, compared with the pursuit of meaning, the presence of meaning is more closely related to the positive psychological quality of individuals.

### Implications

With the continuous development of competitive sports, mental toughness, which is a symbol of athletes' spiritual culture and the crucial psychological advantage of athletes, has increasingly been subject to attention. The results of this study provide strategies for improving the mental toughness of adolescent athletes and helping them maintain high levels of subjective and objective performance under the pressure of training and competition, thus providing practical guidance for the training of adolescent athletes. Specifically, coaches and managers should strengthen the protection and cultivation of adolescent athletes' belief in a just world and the sense of meaning in life in their daily training and life, which will better develop the mental toughness of adolescent athletes. In terms of strategies to maintain and develop adolescent athletes' belief in a just world, Lerner (1980) pointed out that strategies to deal with the threat of unjust situations can mainly be divided into four categories: rational strategy, irrational strategy, protective strategy, and defensive strategy. The rational strategy involves reconstructing justice at a realistic level, which mainly requires preventing the occurrence of unfair events and compensating those who are affected by unfair events after their occurrence. The irrational strategy reconstructs justice at a cognitive level, including the reinterpretation of the attribution of unfair events. Moreover, the protective strategy is a coping strategy from the perspective of worldview construction. It refers to people's belief in ultimate justice, which is used to reevaluate the current injustice, that is, to believe that the world will be just in the long run and that the present injustice will be compensated for by future justice. The defensive strategy is a kind of cynical self-protection, in which people believe that the world is inherently unjust so that it does not matter whether their beliefs in justice are threatened or not, and it is also the last-resort defense mechanism that is considered when all other strategies fail. It is worth noting that the defensive strategy may not be appropriate for adolescent athletes because it can cause a person's belief in a just world to collapse. According to the results of this study, it may lead to the loss of a sense of meaning in life for adolescent athletes, thus compromising their mental toughness. However, the other strategies can be considered to maintain and enhance individuals' belief in a just world from multiple aspects, such as reality and cognition, so as to enhance the sense of meaning in the life of adolescent athletes and promote the development of their mental toughness.

The results of this study broadened the group to which the view is applicable that just-world and life meaning theories can effectively promote individual psychology. In previous studies, belief in a just world and meaning in life were often discussed in relation to stressful situations. Dalbert and Stoeber (2006) reported that the strength of students' belief in a just world was negatively correlated with the stress of their school life. Otto and Schmidt (2007) indicated that the belief in a just world could compensate for the pressure people experience in the workplace and protect their mental health, while Park and Baumeister (2017) proved the connection between people's perception of the meaning of life and their daily stressors. Based on the competitive sports environment, the pressure experienced by Chinese adolescent athletes is unique, continuous, and changing. However, our results show that in this special group of Chinese adolescent athletes, belief in a just world and meaning in life also had positive significance for individual psychological resources such as mental toughness.

### Limitations and Future Research Directions

This study had some limitations: First, it was designed as a cross-sectional study, so it cannot provide accurate causal evidence of a relationship among belief in a just world, meaning in life, and mental toughness. Second, the study only focused on the mediating mechanism of meaning in life in the relationship between belief in a just world and mental toughness, which cannot rule out the existence of other mediating and moderating variables. Third, because this study was conducted on athletes in the context of Chinese culture, it remains to be tested whether the research results are applicable to athletes from other cultural backgrounds. Based on the limitations of the current study, future research could provide strong evidence of a causal relationship between belief in a just world, meaning in life, and mental toughness through longitudinal tracking of the experimental intervention. Future research could also explore other intermediary variables in the relationship between belief in a just world and mental toughness. For example, Callan et al. (2009) once pointed out that when individuals' belief in a just world is damaged more seriously, they will underestimate the value of delayed rewards and be more willing to accept smaller but immediate rewards, instead of larger but delayed rewards. Future studies may test the mediating role of cognitive evaluation between belief in a just world and mental toughness based on this view. In addition, future research could enhance the cultural applicability of the research model structure by investigating athletes from additional cultural backgrounds.

## Conclusion

The mental toughness of adolescent athletes has a profound impact on society and athletes' sports careers, and even on their entire lives. This study introduced just-world and life meaning theories to explore how to improve adolescent athletes' mental toughness. Our significant findings are as follows: First, there were partially demographic differences in the belief in a just world, meaning in life, and mental toughness of adolescent athletes; second, adolescent athletes' belief in a just world was positively associated with mental toughness, suggesting the promoting effect of belief in a just world on mental toughness; third, search for meaning and presence of meaning played a parallel mediating role between belief in a just world and mental toughness in adolescent athletes, and compared with the search for meaning, the presence of meaning had a stronger mediating effect. Clarifying the group differences and the role of belief in a just world and meaning in life on mental toughness can provide ideas for further exploration of the promoting mechanism of adolescent athletes' mental toughness in the future, and guide coaches and managers to design intervention programs on the psychological construction of adolescent athletes.

## Data Availability Statement

The original contributions presented in the study are included in the article/supplementary material, further inquiries can be directed to the corresponding authors.

## Ethics Statement

The studies involving human participants were reviewed and approved by the Ethics Committee of the School of Nursing and Rehabilitation, Shandong University (No. 2022-R-15). Written informed consent to participate in this study was provided by the participants' legal guardian/next of kin. Written informed consent was obtained from the minor(s)' legal guardian/next of kin for the publication of any potentially identifiable images or data included in this article.

## Author Contributions

SC and YC contributed to the conception, design of the study, and critically revised the important intellectual content of the manuscript. YC organized the database. ST and SC performed the statistical analysis. ST wrote the first draft of the manuscript. All authors contributed to manuscript revision, read, and approved the submitted version.

## Conflict of Interest

The authors declare that the research was conducted in the absence of any commercial or financial relationships that could be construed as a potential conflict of interest.

## Publisher's Note

All claims expressed in this article are solely those of the authors and do not necessarily represent those of their affiliated organizations, or those of the publisher, the editors and the reviewers. Any product that may be evaluated in this article, or claim that may be made by its manufacturer, is not guaranteed or endorsed by the publisher.
